# Supplementary material for: Effects of sensorimotor voice training on event-related potentials to pitch-shifted auditory feedback
Source: PLoS One. 2023 Jan 20;18(1):e0269326. doi: 10.1371/journal.pone.0269326 (PMC9858400; doi:10.1371/journal.pone.0269326)
Supplement: Attachment — Submitted filename: Responses to reviewers.docx [file pone.0269326.s002.docx]

We appreciate the editor’s time in providing further suggestions to improve our manuscript (PONE-D-22-14364R1). We have carefully considered each of the comments identified and hopefully have improved the clarity of our manuscript as noted below.

**Additional Editor Comments (Previously Reviewer 1):**

1. Line 40: It would be better to specify how the training methods influenced the brain and vocal responses in the abstract. Did they become larger or smaller after training?

*RESPONSE: Makes sense, we added the requested text:*

Across all training tasks, the N1 and P2 components of the ERPs occurred earlier, and the P2 component of the ERPs occurred with larger amplitude post-training. The voice responses also occurred earlier but with a smaller amplitude due to training.

2. Line 42: "...in the pre-training..." How did the training methods affect the pre-training task? I think only the post-training task can be affected by the training methods.

*RESPONSE: Agreed, we clarified this text.*

3. Line 91: "...modifications..." Can you specify the change of N1 and P2? Were they reduced or enhanced?

*RESPONSE: Certainly, we added the suggested text.*

4. Line 262: You mentioned that up and down responses were aggregated so that the main effect of direction was not tested. However, in Line 337, you reported the main effect of direction (F(1,11) = 5.541, p < 0.05). I wonder which way is correct.

*RESPONSE: Good catch. Direction was significant for voice response latency only, so we went ahead and reported all effects of direction for latency. Direction was not significant for voice amplitude as reported in the methods section so we aggregate across direction for the amplitude findings. We clarified this text.*

5. Line 293-294: It would be better to add standard deviations to the mean values (129.32 ms and 148.94 ms). Same for Line 304 and Line 310.

*RESPONSE: We have now added the standard deviations to the mean values as requested.*

6. Line 305 and Line 313: There was a significant interaction between electrode and group on P2 latency and P2 amplitude. More elaborations should be made here. What is the implication for this interaction?

*RESPONSE: We added the requested text.*

7. Line 320: "...modulated..." Can you specify the change of N1 and P2? Were they reduced or enhanced?.

*RESPONSE: We added the requested text. Specifically, the N1 peak latency and P2 peak latency were reduced, and P2 amplitude was increased from pre-training to post-training across all groups.*

8. Line 347: "...(cite)." Citations should be added here.

*RESPONSE: We included a citation here.*

9. Line 432: "...modified..." reduced or enhanced?

*RESPONSE: We added the requested text: Changes were seen in ERP responses and voice responses, whereby the peak responses occurred earlier and the peak ERP amplitude was enhanced while the peak voice response amplitude was reduced post-training compared to pre-training.*

**Additional comments:**
 1. Both reviewers questioned the proportions of following responses in the pre- and post-training tasks. If the information cannot be retrieved, it, at least, should be mentioned or discussed in the limitations.

*RESPONSE: We added this to the limitations.*

2. The response to Reviewer 1's 5th comment can be added to the main text: the rationale of using 1000-ms long and 200-ms long stimuli.

*RESPONSE: We added the response text.*
